# The Needle‐Free Frontier: Redefining the Limits of Transdermal Insulin Delivery

**DOI:** 10.1002/exp2.70214

**Published:** 2026-08-26

**Authors:** Junge Chen, Yuxuan Liu, Bozhang Xia, Julien Milon Essola, Xing‐Jie Liang

**Affiliations:** ^1^ Beijing Advanced Innovation Center for Biomedical Engineering, School of Engineering Medicine, Beihang University Beijing P. R. China; ^2^ Icahn Genomics Institute, Icahn School of Medicine at Mount Sinai, New York New York USA; ^3^ CAS Key Laboratory For Biomedical Effects of Nanomaterials and Nanosafety, CAS Center for Excellence in Nanoscience, National Center For Nanoscience and Technology of China Beijing P. R. China

## Abstract

Wei and colleagues reported a pH‐responsive, charge‐switchable polyzwitterionic polymer carrier that achieves non‐invasive transdermal insulin delivery without external energy input. The approach leverages adaptive compatibility with the cutaneous microenvironment to shuttle functional macromolecules across intact skin and achieve definitive systemic therapeutic outcomes.

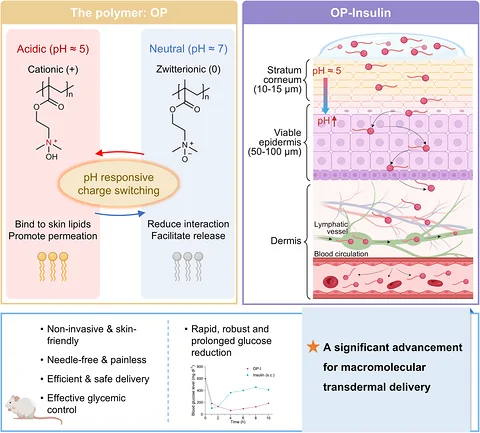

Reducing the treatment burden for patients requiring long‐term therapy is a persistent goal in modern medicine. For the more than 800 million people living with diabetes worldwide, subcutaneous insulin injection remains the standard therapy for type 1 diabetes and advanced type 2 diabetes [1, 2]. However, repeated injections over long periods often cause local side effects, injection‐site pain, lipohypertrophy or lipoatrophy. Moreover, self‐administration at home requires proficient injection technique and strict adherence to the treatment regimen. This poses challenges for elderly patients, children, and others with limited self‐management capacity. As a result, patients with type 2 diabetes who require both basal and prandial insulin therapy often face a substantial treatment burden [3].

Non‐invasive insulin administration has long been pursued in diabetes therapy. However, the skin serves as the body's primary protective barrier. Its outermost layer, the stratum corneum (SC), is a dense matrix approximately 10–15 µm thick, composed of highly ordered lipid lamellae and dehydrated corneocytes, and permits the passive permeation of only a limited range of small‐molecule drugs. Large molecules such as insulin have large molecular weights and complex spatial structures, and are therefore widely considered incapable of achieving effective systemic delivery through intact skin. This pivotal bottleneck has long been an unchallenged consensus in the field for decades.

Against this backdrop, the research team led by Prof. Youqing Shen reported a polyzwitterion, poly[2‐(*N*‐oxide‐*N,N‐*dimethylamino) ethyl methacrylate] (OP), that exhibits pH‐responsive charge‐switching behavior and enables a new strategy for transdermal delivery [1]. The skin is not a homogeneous structure but exhibits an intrinsic pH gradient from the surface to deeper layers. The superficial sebum layer and stratum corneum are mildly acidic (pH ≈ 5), gradually transitioning to a near‐neutral microenvironment toward the viable epidermis and dermis. OP exploits this physiological pH gradient through its pH‐responsive charge‐switching property, thereby enabling transdermal migration of macromolecules across the skin. Under the mildly acidic conditions at the skin surface (pH ≈ 5), the tertiary amine oxide groups of OP become protonated and acquire a positive charge. This facilitates electrostatic interactions with negatively charged lipids in paracellular regions of the stratum corneum, promoting entry of the insulin–polymer complex into the barrier and diffusion along intercellular lipid pathways. As the complex progresses into the viable epidermis and dermis, the local pH gradually increases, triggering deprotonation of OP and conversion to a highly hydrophilic polyzwitterionic state. The lipid interactions are reduced, allowing the complex to enter deeper skin layers. During the transport process through the viable skin layers, OP transiently adsorbs onto cell membranes, and then undergoes contact‐mediated membrane hopping. This process reduces exposure to intracellular enzymes, and allows rapid transit through the viable epidermis and dermis. After crossing the dermis, OP–I enters the cutaneous lymphatic system, and undergoes lymphatic uptake.  Covalent conjugation with OP preserves the binding affinity of insulin for its receptors on target cells. OP–I mainly acts on the systemic insulin receptors in glucose‐regulating tissues, potentially minimizing cutaneous off‐target effects (Figure 1).

**FIGURE 1:**
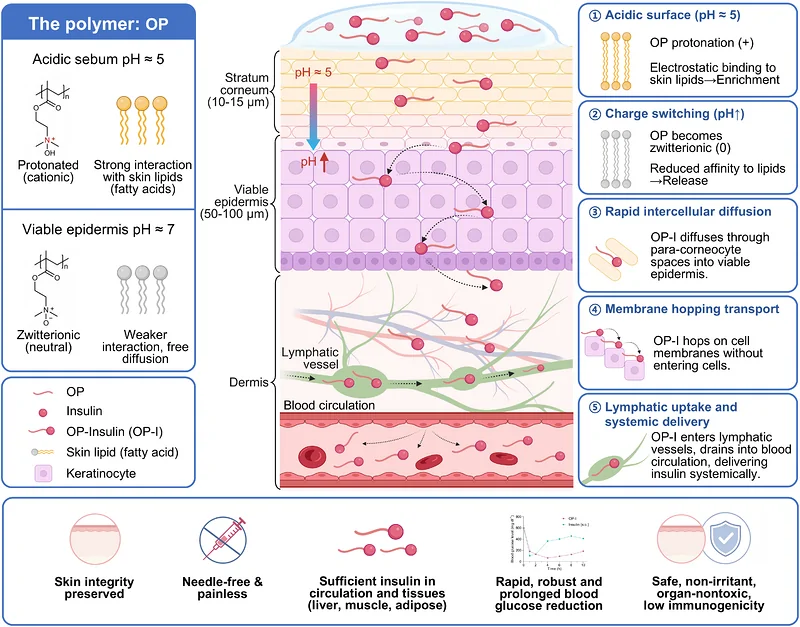
A pH‐responsive polyzwitterion for non‐invasive transdermal insulin delivery to regulate blood glucose, Created in BioRender (https://BioRender.com).

The OP‐mediated transdermal delivery system shows promising translational potential in both in vitro and in vivo studies. In preclinical studies, OP–I is administered topically at a dose of 116 U kg^−1^ to streptozotocin (STZ)‐induced type 1 diabetic mice, which reduced blood glucose levels to below 200 mg dL^−1^ within 1 h, and maintained normoglycemia (50–200 mg dL^−1^) for up to 12 h [1]. By comparison, subcutaneous injection of insulin at 5 U kg^−1^ caused blood glucose to rebound to roughly 400 mg dL^−1^ after 4 h. Notably, OP–I displayed a hypoglycemic effect similar to subcutaneous insulin, but provided more sustained glycemic control, which might reduce glucose fluctuations. In the case of diabetic minipigs, an animal model with skin architecture closely resembling human skin, the topical application of OP–I at a dose of 29 U kg^−^^1^ was performed. This application reduced blood glucose to normoglycaemic levels within 2 h, and it maintained therapeutic effect for approximately 12 h. Unlike the conventional chemical penetration enhancers, which disrupt the skin barrier, the repeated administration of OP–I did not affect the structural integrity of the stratum corneum, and also did not induce any detectable local inflammatory responses. These findings indicate a favorable biosafety.

Wei et al. introduce and validate a biomacromolecule delivery strategy that exploits the physiological gradients of the skin [1]. By demonstrating that macromolecules can be transported across intact skin without invasive intervention, the work challenges the long‐standing view that non‐invasive transdermal delivery of biomacromolecules is infeasible. It therefore provides a conceptual and practical framework for rethinking administration strategies for protein and peptide therapeutics. The transdermal strategy enhances convenience and adherence, providing a foundation for home‐based diabetes management. Future work needs to evaluate skin tolerability under chronic use in animal models, optimize dosage forms (e.g., patches, wearable devices), integrate continuous glucose monitoring, and account for cutaneous physiological variations (aging, obesity) to enable individualized dosing.

Beyond insulin, this carrier demonstrates broad applicability for diverse macromolecules including liraglutide, semaglutide, proteins, antibodies, and siRNA. By achieving deep dermal penetration without compromising skin integrity, the platform may extend to localized bioactive delivery (e.g., growth factors for chronic wounds) while reducing invasive procedures. It further offers an alternative to long‐term injections for chronic diseases. Defining the full scope of this platform and accelerating its clinical translation will require integrating macromolecular physicochemical properties with skin microenvironment dynamics.

Overall, amid global population aging and escalating demand for home‐based long‐term therapies, this work makes a significant breakthrough in macromolecular transdermal delivery, offering new hope for non‐invasive diabetes treatment. By achieving effective glycemic control through simple topical application—liberating patients from needle dependence—it overturns the prevailing assumption that functional macromolecular therapeutics require invasive or externally assisted delivery strategies. This paradigm shift transcends mere alleviation of physical pain, fundamentally restoring patient dignity and enhancing quality of life. Through systematic translation of an original conceptual breakthrough from inception to validation, this technology has also been successfully applied to the transdermal delivery of other biological macromolecules such as peptides, proteins and nucleic acids, setting a new benchmark for the development of global non‐invasive biological macromolecule delivery technology.

## Funding

This work was supported by the Beijing Nova Program (202604841317).

## Conflicts of Interest

Xing‐Jie Liang is a member of the Exploration editorial board, and he was not involved in the handling process of this manuscript.

## Data Availability

The data that support the findings of this study are available from the corresponding author upon reasonable request.
